# Incidence of malignant neoplasms among HIV-infected persons in Scotland

**DOI:** 10.1038/sj.bjc.6601139

**Published:** 2003-07-29

**Authors:** G M Allardice, D J Hole, D H Brewster, J Boyd, D J Goldberg

**Affiliations:** 1 Department of Statistics and Modelling Science, University of Strathclyde, 7th Floor Livingstone Tower, 26 Richmond Street, Glasgow G1 1XH, UK; 2 Department of Public Health, University of Glasgow, Glasgow G12 8RZ, UK; 3 Information and Statistics Division, Common Services Agency, Trinity Park House, Edinburgh EH5 3SQ, UK; 4 Scottish Centre for Infection and Environmental Health, Clifton House, Clifton Place, Glasgow G3 7LN, UK

**Keywords:** HIV, AIDS, cohort study, linkage, Scotland

## Abstract

Among 2574 persons diagnosed with HIV throughout Scotland and observed over the period 1981–1996, cancer incidence compared to the general population was 11 times higher overall; among homosexual/bisexual males, it was 21 times higher and among injecting drug users, haemophiliacs and heterosexuals it was five times higher, mostly due to AIDS-defining neoplasms. However, liver, lung and skin cancers (all non-AIDS-defining) were also significantly increased.

Knowledge of cancer excess in people with HIV has largely been based on (a) case reports or case series (b) matching population-based cancer registries with AIDS registries or (c) following up specific cohorts of HIV-infected individuals. Large relative risks are reported for AIDS-defining cancers, but the possible relationship between HIV and non-AIDS-defining cancers is less conclusive. In Scotland, a high incidence of many cancers and the existence of two high-quality, national, computerised databases on HIV and cancer incidence afforded an opportunity to adopt a computer linkage approach to examine the occurrence of malignant neoplasms (both AIDS and non-AIDS defining) among all HIV–diagnosed persons across Scotland.

## MATERIALS AND METHODS

Records on Scotland's HIV database were linked with those on the Scottish Cancer Registry to determine the incidence of malignant neoplasms among persons diagnosed with HIV in Scotland. The Scottish HIV Database (Goldberg *et al*, 1992) holds data on all persons who have tested HIV antibody positive and/or been diagnosed with AIDS in Scotland since the early 1980s; data include date of birth, initials, soundex code of surname, AIDS-defining illness and dates of first HIV antibody positive test, AIDS diagnosis and death. The risk category distribution of the database's diagnoses is relatively even: 34% injecting drug users (IDU), 34% homosexual/bisexual males, 24% heterosexual and 8% other risk groups. The Scottish Cancer Registry holds a range of both patient and tumour-related information, including demographic and diagnostic details, on all cancers (ICD-9 codes 140–208) diagnosed in Scotland since 1959.

The linkage exercise was restricted to the period 1980–1996, the era prior to the full introduction of Highly Active Antiretroviral Therapy (HAART) in Scotland. All malignant neoplasms identified through the linkage process were eligible for inclusion in the analysis, provided they were diagnosed after the HIV diagnosis or preceded it by 2 months or less. This latter criterion allowed for the possibility that the discovery of a malignant neoplasm may have instigated an HIV test and subsequent HIV diagnosis. Links between the two databases were identified using probability linkage techniques and utilising the expertise of the Scottish Record Linkage Group (Kendrick and Clarke, 1993).

The expected numbers of malignant neoplasms occurring among the HIV cohort, assuming the same incidence as that for a matched general population in Scotland, was calculated using age-group, sex, year (for the period 1980–1996) and site-specific data held on the Scottish Cancer Registry. The Standardised Incidence Ratio (SIR) was defined as the ratio of the observed to expected number of neoplasms and the confidence interval calculated assuming that the observed number followed a Poisson distribution.

## RESULTS

The HIV Database comprised 2574 HIV-infected individuals testing HIV antibody positive between 1981 and 1996, of whom 1955 were male and 619 female. Follow-up to death or 31st December 1996 provided 11 677 and 4236 person-years of follow-up for males and females, respectively. The cohort's median age at HIV diagnosis was 28 years and comprised 58 children (aged 14 years or less at HIV diagnosis). The linkage process identified 162 malignant neoplasms (two occurring in children). The incidence of malignancy among the HIV-infected population was 11 times that of the general population, SIR=10.8 (95% CI 9.2, 12.6). The incidence was 21 times higher among homosexual/bisexual males and five to six times higher among the IDU, haemophiliac and heterosexual risk groups (Table 1

**Table 1:** Observed malignant neoplasms by HIV Risk Group, AIDS-defining status, site and morphology: SIR and 95% CI (SIR)

**HIV risk group**	**AIDS/non-AIDS defining**	**Cancer site**	**Morphology**	***N***	**SIR**	**95% CI SIR**
All risks	AIDS and non-AIDS	All	All	162	10.8	9.2, 12.6
Heterosexual	AIDS and non-AIDS	All	All	14	5.9	3.2, 9.9
Homo/bisexual	AIDS and non-AIDS	All	All	102	21.4	17.4, 26.1
IDU	AIDS and non-AIDS	All	All	35	5.5	3.8, 7.6
Haemophiliac	AIDS and non-AIDS	All	All	5	6.1	2.0, 14.2
Other/mixed	AIDS and non-AIDS	All	All	6	9.0	3.3, 19.6
						
All risks	AIDS	All	All	138	100	84.1, 118.1
All risks	AIDS	Skin (45), Connective and soft tissue (5) Head and Neck (4) Lung (1)	Kaposi's sarcoma	55	2750	2067, 3583
All risks	AIDS	Not available	Non-Hodgkin's lymphoma	82	108	85.6, 134.4
All risks	AIDS	Cervical	Squamous cell carcinoma	1	1.7	0.04, 9.26
						
All risks	Non-AIDS	All	All	24	1.8	1.1, 2.6
All risks	Non-AIDS	Skin	Squamous cell carcinoma (3), Basal cell carcinoma (1), Unspecified (2)	6	2.8	1.04, 6.2
All risks	Non-AIDS	Lungs	Squamous cell carcinoma (1), Small-cell carcinoma (1), Adenocarcinoma (2), Unspecified (1)	5	4.1	1.3, 9.5
All risks	Non-AIDS	Liver	Hepatocellular carcinoma	2	22.0	2.7, 80.2
All risks	Non-AIDS	Bladder	Transitional cell carcinoma (1), Papillary cell carcinoma (1)	2	4.2	0.5, 15.0
All risks	Non-AIDS	Brain and CNS	Astrocytoma	2	3.3	0.4, 12.0
All risks	Non-AIDS	Not available	Hodgkin's disease	2	3.6	0.4, 13.1
All risks	Non-AIDS	Bone	Ewing's sarcoma	1	9.1	0.23, 50.5
All risks	Non-AIDS	Head and neck	Unspecified	1	1.6	0.04, 8.8
All risks	Non-AIDS	Leukaemias	Acute myeloid leukaemia	1	2.2	0.06, 12.4
All risks	Non-AIDS	Stomach	Unspecified	1	2.9	0.07, 15.9
All risks	Non-AIDS	Testis	Seminoma	1	0.7	0.02, 3.75

). Of the 138 AIDS-defining malignant neoplasms, 82 were non-Hodgkin's lymphomas, 55 Kaposi's sarcomas and one was an invasive cervical cancer. The SIRs for the 138 AIDS-defining and 24 non-AIDS-defining neoplasms were 100.0 (95% CI 84.1, 118.1) and 1.8 (95% CI 1.1, 2.6), respectively. Most of the non-AIDS-defining SIRs were of the order 1–4, with the exception of those for liver and bone cancers. The SIRs for liver, lung and skin cancers were statistically significant.

## DISCUSSION

This study confirmed the large expected excess risks of non-Hodgkin's lymphoma and Kaposi's sarcoma among HIV-infected persons; the latter finding is consistent with the previous observation of an increased incidence of Kaposi's sarcoma during the mid 1980 s in Scotland (Brewster *et al*, 1999). Although invasive cervical cancer is an AIDS indicator disease, only one case was diagnosed; its SIR was 1.7 (0.04, 9.26), a considerably lower ratio than the statistically significant ones associated with cancers of the liver and lungs.

An important finding was the SIR of 1.8 (95% CI 1.1, 2.6) for the non-AIDS-defining malignant neoplasms. Other studies identified a 2-3 fold risk of non-AIDS-defining malignant neoplasms among AIDS patients and an excess of liver cancer (Serraino *et al*, 2000), lung cancer (Grulich *et al*, 1999; Frisch *et al*, 2001) and skin cancers (Cooksley *et al*, 1999) among HIV-infected persons. Unlike several other studies, we did not find a significant excess of Hodgkin's disease (Cooksley *et al*, 1999; Grulich *et al*, 1999; Serraino *et al*, 2000; Frisch *et al*, 2001).

The possibility that some of the non-AIDS-defining neoplasms were extranodal lymphomas, or Kaposi's sarcomas that had been misdiagnosed or miscoded as other types of cancer, cannot be excluded. Most of the cancers in question, however, were recorded as being microscopically verified and the reliability of diagnostic coding for the Scottish Cancer Registry is high (Brewster *et al*, 1994).

In this study, it was impossible to associate cancers with immunodeficiency, lifestyle, deprivation or coinfection with another virus. Accordingly, the excess of lung cancer may be due to high rates of smoking among HIV-infected gay men and the excess of liver cancer may be related to HIV/hepatitis coinfection, especially among IDUs. In Scotland, the incidence of many cancers varies by deprivation category; however, deprivation scores were not available for the HIV cohort.

The study method is subject to some potential biases. Persons belonging to the HIV cohort might have been subject to closer clinical scrutiny than those in the general population; this could have led to earlier diagnoses in the former. In contrast, emigration of any HIV-infected persons will have tended to underestimate cancer risk due to an overestimation of person-years at risk and the failure to ascertain and register any subsequent cancers arising in emigrants.

Since the aim of the investigation was to determine the natural history of neoplasia incidence among a national HIV cohort, the linkage was not extended beyond 1996, the time when Highly Active Anti-Retroviral Therapy (HAART) became widely administered in Scotland. The investigators plan to repeat the linkage to determine how much the incidence of neoplasia has changed during the era of HAART.
